# Identification and characterization of preferred DNA-binding sites for the *Thermus thermophilus* transcriptional regulator FadR

**DOI:** 10.1371/journal.pone.0184796

**Published:** 2017-09-13

**Authors:** Minwoo Lee, Hyejin Um, Michael W. Van Dyke

**Affiliations:** Department of Chemistry and Biochemistry, Kennesaw State University, Kennesaw, Georgia, United States of America; Universite Paris-Sud, FRANCE

## Abstract

One of the primary transcriptional regulators of fatty acid homeostasis in many prokaryotes is the protein FadR. To better understand its biological function in the extreme thermophile *Thermus thermophilus* HB8, we sought to first determine its preferred DNA-binding sequences *in vitro* using the combinatorial selection method Restriction Endonuclease Protection, Selection, and Amplification (REPSA) and then use this information to bioinformatically identify potential regulated genes. REPSA determined a consensus FadR-binding sequence 5´-TTRNACYNRGTNYAA-3´, which was further characterized using quantitative electrophoretic mobility shift assays. With this information, a search of the *T*. *thermophilus* HB8 genome found multiple operons potentially regulated by FadR. Several of these were identified as encoding proteins involved in fatty acid biosynthesis and degradation; however, others were novel and not previously identified as targets of FadR. The role of FadR in regulating these genes was validated by physical and functional methods, as well as comparative genomic approaches to further characterize regulons in related organisms. Taken together, our study demonstrates that a systematic approach involving REPSA, biophysical characterization of protein-DNA binding, and bioinformatics can be used to postulate biological roles for potential transcriptional regulators.

## Introduction

Genome projects have yielded considerable information since the sequencing of the first whole microorganism genome, *Haemophilus influenza*, in 1995 [1,2]. However, beyond a mere identification of open reading frames, it is important to determine the biological functions of encoded proteins and RNAs. One subset of proteins eliciting considerable interest is transcription factors, sequence-specific DNA-binding proteins that regulate transcription initiation, a major means of regulating gene expression. In prokaryotic organisms, genes encoding transcription factors are estimated to constitute, on average, ~5% of all protein-coding genes [3,4]. This reflects the need for prokaryotes to respond to a variety of changes in their environment necessitating a tight level of control over the expression of specific sets of genes, including additional transcription factors as part of a regulatory network. For a well-characterized organism such as *Escherichia coli*, 304 of its 4140-identified protein-coding genes are postulated to encode for transcription factors [5]. Of these, detailed DNA binding information (*e*.*g*., position-specific scoring matrices or sequence logos) is available for just over half. Such is even more apparent for the less well-characterized extremely thermophilic, aerobic eubacteria *Thermus thermophilus* HB8, where of its 2173 identified protein-coding genes, ~70 are predicted to be transcription factors and detailed DNA binding information is available for only a handful [6–15]. Increased knowledge of transcription factors and the genes they control will be essential in furthering the understanding not only of an organism’s regulatory networks but also its fundamental biology and relationship with its environment.

Transcription factors are typically first identified in genomic screens by their protein sequence similarity with known transcription factors, particularly with regards to their DNA-binding domains [16]. Beyond that, further characterization then requires identifying the set of genes they regulate. In organisms with tractable genetics, this can be achieved through the construction of a transcription factor deletion strain and by then comparing the levels of RNA present in wild-type and deletion strains for all possible transcripts [17,18]. However, such genetics are not always feasible in all organisms and microarray-based surveys can be prone to false positives (*e*.*g*., gene targets of downstream transcription factors) and false negatives (*e*.*g*., low transcript levels). Thus, approaches based on determining the intrinsic DNA-binding specificity of a transcription factor and mapping these sites to the organism genome can be used to identify potential regulated genes. While transcription factor DNA-binding specificity is often determined by comparing gene promoters identified in genetic/microarray screens and finding regions of sequence similarity, a more *a priori* determination can be achieved by using combinatorial selection methods, *e*.*g*., CASTing, SELEX and SAAB [19–21]. Our laboratory has developed an alternative combinatorial selection approach, Restriction Endonuclease Selection Protection and Amplification (REPSA), that relies on the ligand-dependent protection of PCR templates from enzymatic inactivation by type IIS restriction endonucleases (IISRE), which cleave double-stranded DNA without sequence specificity at a fixed distance from their recognition sequence [22]. We have successfully used REPSA to identify the preferred DNA-binding sequences of triplex-forming oligonucleotides, transcription factors, and various small molecules important in cancer chemotherapy [23–28]. More recently we have used REPSA for the discovery of DNA-binding proteins involved in nucleoid exclusion and transcription regulation [29,30].

*T*. *thermophilus* HB8 FadR is a 205-amino acid protein encoded by the TTHA0101 gene *fad*R, with an expected molecular mass of 23,620 Da. It contains a predicted TetR-type α-helix-turn-α-helix (HTH) motif from amino acids 9–69 and would be expected to bind a palindromic DNA sequence as a homodimer. This protein has been investigated as part of the Structural and Functional Whole Cell Project and two crystal structures presently exist [7,31]. These investigators also compared mRNA levels from Δ*fadR* and wild-type *T*. *thermophilus* HB8 strains to identify FadR-regulated genes, validated transcriptional regulation *in vitro* on nine promoters, and characterized the FadR-DNA binding properties on one [7]. From these studies, they identified a putative *T*. *thermophilus* FadR-binding site 5´-TTANACT-(N_6-7_)-ARNNNAR-3´ and five operons (*TTHA0103–0101*, *TTHA0401–0400*, *TTHA0890–0892*, TTHA*1144–1146*, and TTHB*017–012*), and four individual genes (*TTHA0604*, *TTHA0846*, *TTHA1117*, and *TTHA1463*) regulated by FadR. Notably, many of these genes are homologs of known FadR-regulated genes in *Escherichia coli* or *Bacillus subtilis* that encode for proteins involved with fatty acid degradation [32,33].

In the present report, we describe the application of REPSA to determine the preferred DNA-binding sequences for the *T*. *thermophilus* HB8 transcription factor FadR. Our studies yielded a 15-mer FadR-binding consensus sequence with high significance. Mapping the FadR consensus sequence to the *T*. *thermophilus* HB8 genome identified several promoter regions capable of binding FadR. These were found to correspond to operons encoding proteins involved with fatty acid homeostasis as well as other biological processes, thus providing insights into the biological function of FadR in *T*. *thermophilus*.

## Results

### FadR expression and characterization

*E*. *coli* strain BL21(DE3), transformed with the plasmid pET11a-ttfadR, was used to express the *T*. *thermophilus* HB8 FadR protein. Following induction, whole cell extracts were prepared and then heat-treated to denature *E*. *coli* proteins. Given the thermostability of FadR, it remained soluble, allowing the facile removal of most contaminating *E*. *coli* proteins by centrifugation. This purified FadR preparation was found to contain a single dominant protein species of apparent molecular masses 21-kDa as indicated by SDS-PAGE (S1 Fig, Panel A, lane 4). Densitometric quantitation indicated that FadR was approximately 90% pure. However, an Agilent P200 ScreenTape assay found the major 20.6 kDa species corresponding to FadR constituted only 59.24% of all integrated area in the run, suggesting lower purity (S1 Fig, Panel C). Given that the stock FadR had a protein concentration of 0.7 mg/ml, we estimate that its concentration was no greater than 30 μM FadR monomer.

### REPSA selection of FadR-binding DNAs

**Fig 1:**
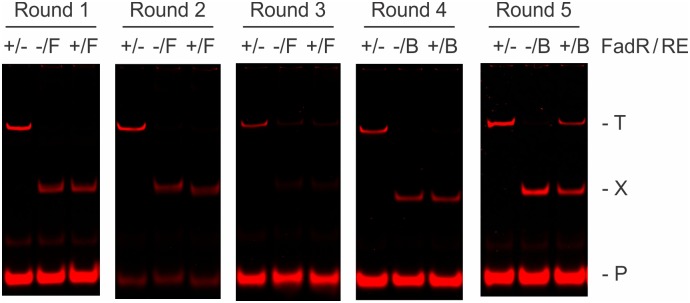
REPSA section of FadR-dependent IISRE cleavage-resistant DNA species. Shown are LICOR Odyssey images of restriction endonuclease cleavage protection assays during Rounds 1 through 5 of REPSA selection with 6 nM FadR protein. The presence of FadR or IISRE FokI (F) or BpmI (B) is indicated above each lane. Lanes include: (+/-) total DNA control, (-/F or -/B) IISRE cleavage control, and (+/F or +/B) IISRE selection with FadR. The electrophoretic mobility of the intact (T) and cleaved (X) selection template, as well as the IRD7_ST2R primer (P), are indicated at right of figure.

Our standard selection template ST2R24, containing recognition sites for IISREs FokI and BpmI and a 24-bp randomized sequence, was used in the REPSA selection of FadR binding sequences [30]. Starting population was 42 fmoles or 2.5 × 10^10^ molecules of ST2R24 DNA, which provides sufficient coverage to investigate the combinations possible for a 16-bp recognition sequence (2.1 × 10^9^). A total of five rounds of REPSA were performed, three with the IISRE FokI and two with BpmI. This change in IISRE was necessitated by the appearance of FadR-independent, FokI cleavage resistance (Fig 1, Round 3), a phenomenon that has been noted previously [23,30]. Evidence for a FadR-dependent, IISRE cleavage-resistant DNA population was observed at Round 5. While incomplete, this level of IISRE protection is comparable with prior REPSA studies and has been found indicative of a majority of DNAs possessing preferred ligand-binding sites [23,26–28,30].

**Fig 2:**
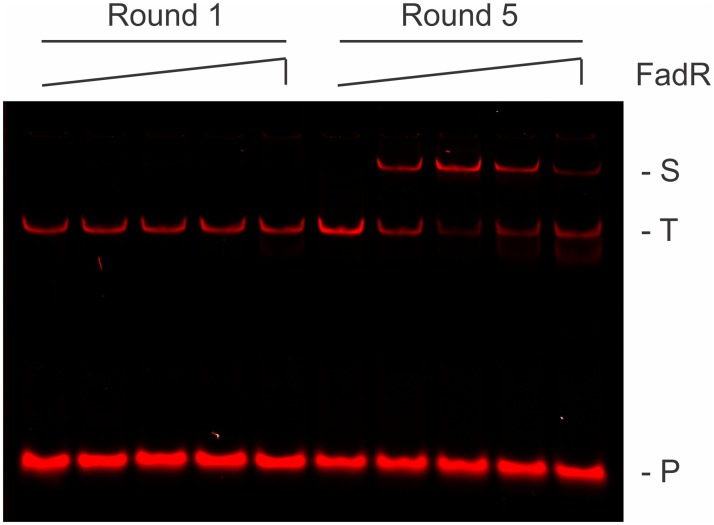
Validation of REPSA-selected FadR-binding DNA species. Shown are LICOR images of EMSAs containing pooled DNA from either Round 1 (left lanes) or Round 5 (right lanes) of REPSA selection and increasing concentrations of FadR protein (from left to right: 0, 0.6, 6, 60, or 600 nM FadR). The electrophoretic mobility of a single protein-DNA complex (S) as well as uncomplexed ST2R24 selection template (T) and IRD7_ST2R primer (P) are indicated at right of figure.

Before massive parallel sequencing, the presence of FadR-binding sites within the REPSA selected DNAs was first validated using an electrophoretic mobility shift assay (EMSA). 7.4 fmoles of PCR DNA product from either Round 1 or Round 5 was incubated with increasing concentrations of FadR protein under conditions to permit specific DNA binding. As shown in Fig 2, no evidence for FadR-DNA complexes was observed with Round 1 DNA, even at high (600 nM) FadR concentrations. This indicates that FadR does not form electrophoretically stable complexes with nonspecific DNAs under these reaction conditions. However, with Round 5 DNA, a substantial percentage of the DNA was present in a single slower mobility species, even at concentrations as low as 0.6 nM FadR. This was considered good evidence that the majority of the Round 5 DNAs contained stable, high-affinity FadR-binding sites and was worthy of massive parallel sequencing. Curiously, the greatest extent of FadR-DNA complex formation was observed at intermediate (6 nM) and not at the highest (600 nM) FadR concentration. This unexpected result was found to be reproducible but only with this mixed population of DNA (data not shown). The cause for this phenomenon remains to be determined.

### Sequencing and motif elucidation of REPSA-selected, FadR-binding DNA

DNA from Round 5 REPSA selection was used to generate a fusion amplicon library suitable for semiconductor-based sequencing using an Ion Personal Genome Machine (Ion PGM). Sequencing yielded 2,070,000 total bases, 1,523,020 ≥ *Q*20, and resulted in 41,003 reads of 50 bp mean read length. A fastq format data file was generated from this run and further processed by our Sequencing1.java program to yield data in a format suitable for input into MEME Suite analysis software [34]. For FadR-selected DNA, processing yielded 5,005 sequences. Of these, one was found in triplicate and 13 in duplicate, giving 4,990 unique sequences.

**Fig 3:**
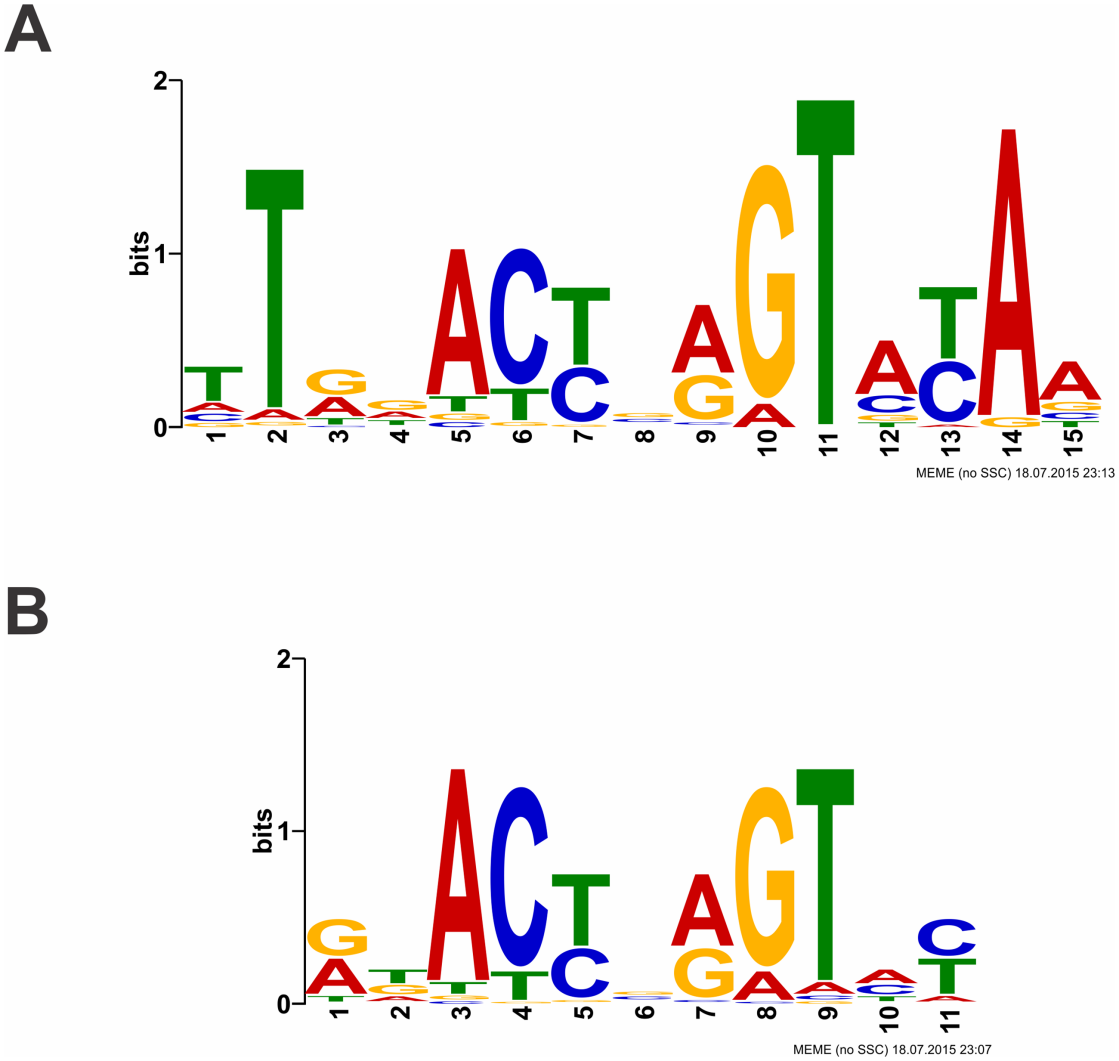
Sequence logos of REPSA-selected FadR-binding sequences. Sequence logos were determined using MEME software with inputs of 1000 Round 5 DNA sequences. (**A**) MEME performed with no filters. (**B**) Palindromic filter.

Web version 4.10.2 of Multiple Em for Motif Elicitation (MEME) was used to discover protein binding motifs in the Round 5 REPSA-selected DNA sequences. Input was the first 1000 sequences obtained from the Sequencing1.java output file, the maximum number accommodated by MEME. A nonpalindromic MEME analysis identified a single 15-mer motif that was present in the vast majority of the sequences (849/1000). The statistical significance of this motif, as measured by its *E*-value, was 3.6e-1789. A sequence logo of its position weighted matrix is shown in Fig 3A. As most TetR-family HTH proteins exist as homodimers and recognize palindromic binding sites, we repeated the MEME analysis with a limit to palindromic sequences advanced option. This analysis found a 11-mer motif (Fig 3B) in 929/1000 sequences with an *E*-value of 2.9e-1102. Interestingly, the palindromic sequence logo contained sequences from the center of the nonpalindromic sequence logo, with slight differences. From these two motifs, a 15-mer pseudopalindromic consensus sequence was derived, 5´-tTRNACYNRGTNYAa-3´, where bases in lowercase were deemed less significant in determining specific FadR-DNA binding. Note that while additional MEME analyses were performed with subsequent sets of 1000 sequences, in all cases, very similar results were obtained (data not shown). Taken together, these analyses strongly suggest that the derived consensus sequence corresponds to a high-affinity FadR binding sequence, and that those bases most prominently represented in these sequence logos represent those individual bases that are most important in FadR-DNA recognition.

### Characterization of REPSA-identified, FadR-binding sequences

**Fig 4:**
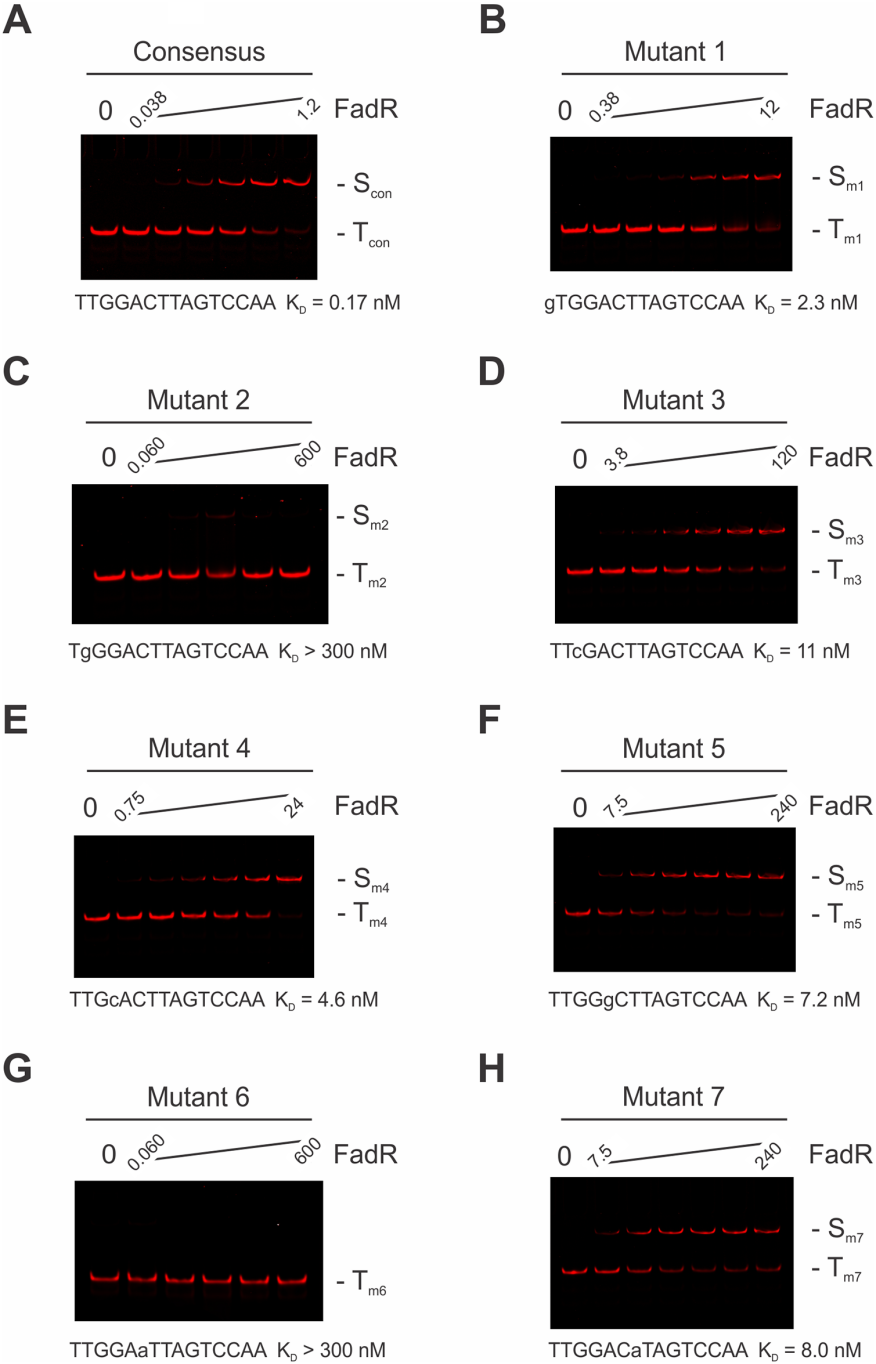
Quantitative EMSA analysis of FadR-binding to consensus and mutant sequences. Shown are LICOR images of IRD700-labeled FadR consensus or point-mutated DNAs, as indicated, incubated with a twofold (wt, m1, m3, m4, m5, m7) or tenfold (m2, m6) titration of FadR protein, as indicated. (S) FadR-DNA complex, (T) uncomplexed DNA. (**A**) ST2_FadR_R5_wt consensus DNA; 0, 0.038, 0.075, 0.15, 0.3, 0.6, 1.2 nM FadR. (**B**) ST2_FadR_R5_m1 mutant DNA; 0, 0.38, 0.75, 1.5, 3.0, 6.0, 12 nM FadR. (**C**) ST2_FadR_R5_m2 mutant DNA; 0, 0.06, 0.6, 6, 60, or 600 nM FadR. (**D**) ST2_FadR_R5_m3 mutant DNA; 0, 3.8, 7.5, 15, 30, 60, 120 nM FadR. (**E**) ST2_FadR_R5_m4 mutant DNA; 0, 0.75, 1.5, 3.0, 6.0, 12, 24 nM FadR. (**F**) ST2_FadR_R5_m5 mutant DNA; 0, 7.5, 15, 30, 60, 120, 240 nM FadR. (**G**) ST2_FadR_R5_m6 mutant DNA; 0, 0.06, 0.6, 6, 60, or 600 nM FadR. (**H**) ST2_FadR_R5_m7 mutant DNA; 0, 7.5, 15, 30, 60, 120, 240 nM FadR. Binding site sequence and K_D_ values are indicated below each panel. Lowercase nucleotides indicate mutation from consensus FadR sequence.

To better understand the binding specificity of FadR, quantitative electrophoretic mobility shift assays (EMSA) were performed with DNA probes containing the 15-mer FadR consensus, 5´-TTGGACTTAGTCCAA-3´, or singly point-mutated sequences that contained the least represented base from the position weight matrices at each position within the left half of the pseudopalindromic FadR consensus. Exact sequences may be found in S1 Table. Initial EMSA experiments were performed through a broad range of FadR concentrations (0.06 to 600 nM) to provide a rough estimate of the FadR concentration necessary to observe 50% FadR-DNA complex formation. Final EMSA experiments were performed through a finer, 32-fold range of FadR concentrations, to better estimate FadR concentration at this midpoint. Examples of these experiments are shown in Fig 4. Quantitation of this data was then performed through a densitometric analysis of the IR fluorescence images and approximate the K_D_ for the FadR-DNA complexes determined using an equilibrium binding model. These data are presented in Fig 4. From this study, we found that the consensus FadR sequence had an apparent dissociation constant of 0.17 nM, while mutants m1, m3, m4, m5, and m7 were in the 2–11 nM range. FadR-binding for mutants m2 and m6 were estimated to be greater than 300 nM, the maximum concentration investigated. Taken together, our data show that the electrophoretic stability of FadR-DNA complexes was acutely sensitive to sequence, with single point mutations resulting in 10- to > 1500-fold decreases in stability.

### Identification of potential FadR-binding sites within the *T*. *thermophilus* genome

**Fig 5:**
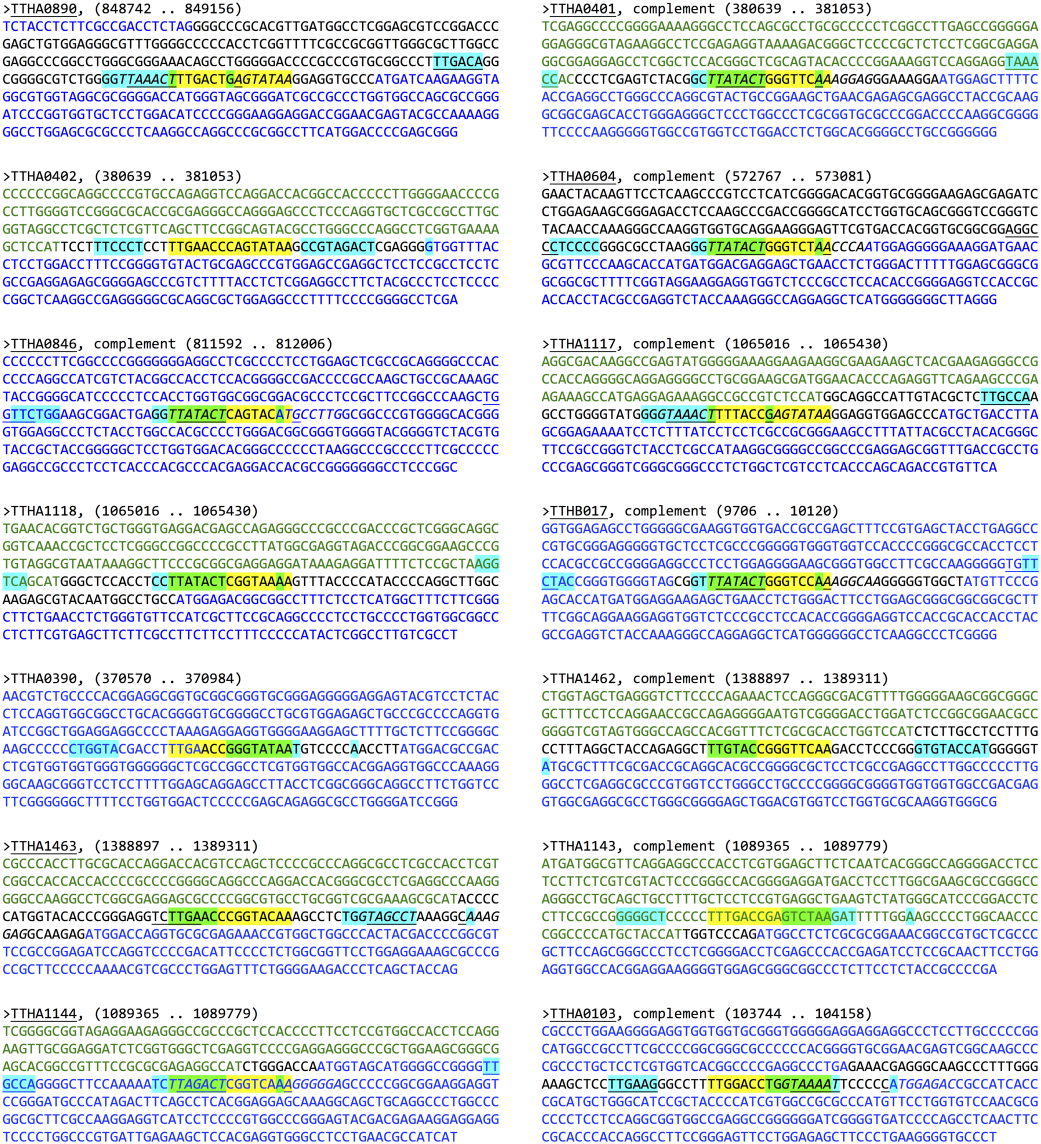
Bioinformatic identification of *T*. *thermophilus* HB8 promoters potentially regulated by FadR. Shown are sequences +/- 200 bp of the FadR-binding sequence of a target gene identified through FIMO analysis as being potentially regulated by FadR (see Table 1). Longest open reading frames with identical orientation as the target gene are indicated with blue nucleotides. Open reading frames with opposite orientation are indicated with green nucleotides. Black nucleotides indicate intergenic regions. Potential promoter elements (-30 and -10 boxes, +1 start site of transcription) were identified using Softberry BPROM and are indicated with blue highlighting. FadR-binding sites are indicated with yellow highlighting. Regions of overlap between FadR-binding sites and promoter elements are indicated by green highlighting. Underlining and italics indicate core promoter elements and FadR-binding sites, respectively, identified previously by Agari *et al* [7].

Using the MEME Suite program Find Individual Motif Occurrences (FIMO), the 15-mer FadR consensus sequence was used to probe the GenBank *Thermus thermophilus* HB8 uid13202 version 210 database using default parameters. Output was 508 motif occurrences with a *P*-value being less than 0.0001. The top 16 occurrences, those whose *P*-value was < 7.0 × 10^−7^ and *Q*-value, a measure of false discovery rate, was < 0.14, were then subjected to further evaluation. These cutoffs were chosen given our experiences with other *T*. *thermophilus* HB8 transcription regulators [30]. Table 1 shows a list of these, removing duplicates that map for the same gene. These sequences were then mapped by hand to their corresponding sites within the *T*. *thermophilus* HB8 genome (KEGG T00220, ttj), to identify genes/operons that could potentially be regulated by FadR. Notably, of the top 16 sites chosen, 14 were located proximal to the postulated start site of translation for identified genes, suggesting they could have FadR involved in their regulation. For these sites, sequences ±200 bp of the genomic FadR site was analyzed using both Softberry BPROM and University of Groningen PePPER to identify potential promoters [35,36]. Although these programs are trained using *E*. *coli* or *Bacillus* and related strain promoters, respectively, they provide the best available tools to identify potential *T*. *thermophilus* core promoter elements. Those 14 sites with high scoring promoters are indicated in Table 1 and mapped with the putative FadR-binding sequence in Fig 5. For the potential FadR-regulated genes, all demonstrated FadR binding sites that were overlapping and/or within their identified core promoter regions. These findings suggest that FadR could transcriptionally regulate these genes.

**Table 1:** FIMO of best possible match TTGGACTTAGTCCAA.

Start	End	*P*-value	*Q*-value	Sequence	Location	Pro?	Gene
848942	848956	6.01e-10	0.00183	TTTGACTGAGTATAA	–11	Y	*TTHA0890*
380839	380853	1.66e-09	0.00183	TTGAACCCAGTATAA	–14	Y	*TTHA0401*
					–17	Y	*TTHA0402*
572967	572981	1.66e-09	0.00183	TTAGACCCAGTATAA	–5	Y	*TTHA0604*
811792	811806	1.73e-09	0.00183	ATGTACTGAGTATAA	+2	Y	*TTHA0846*
1065216	1065230	3.39e-09	0.00287	TTTTACCGAGTATAA	–14	Y	*TTHA1117*
					–47	Y	*TTHA1118*
9906	9920	4.48e-09	0.00316	TTGGACCCAGTATAA	–17	Y	*TTHB017*
370770	370784	8.38e-09	0.00507	TTGAACCGGGTATAA	–14	Y	*TTHA0390*
1389097	1389111	5.04e-08	0.0213	TTGAACCCGGTACAA	–26	Y	*TTHA1462*
					–36	Y	*TTHA1463*
1549819	1549833	7.54e-08	0.0249	CTGTACTCGGTATAA	+23	~	*TTHA1634*
1089565	1089579	8.10e-08	0.0249	TTTGACCGAGTCTAA	–52	Y	*TTHA1143*
					+42	Y	*TTHA1144*
103944	103958	6.68e-07	0.135	TTGGACCTGGTAAAA	–8	Y	*TTHA0103*
724515	724529	6.99e-07	0.135	CTGGACTTGGTCTAA	+909	N	*TTHA0758*

(*P*-value) Defined as the probability of a random sequence of the same length matching that position of the sequence with as good or better score. (*Q*-value) False discovery rate if the occurrence is accepted as significant. (Location) Distance of the FadR binding site from the identified start site of translation. (Pro?) Promoter identified proximally upstream of the gene.

^(~)^ Indicates that while a promoter is present, the FadR-binding site does not overlap.

### Identification of potential *T*. *thermophilus* FadR-regulated genes

As many prokaryotic genes are regulated as members of operons, an analysis of operon structure in the vicinity of the 14 identified genomic FadR binding sites described in Fig 5 was undertaken. Operons identified in the databases provided by the National Autonomous University of Mexico (ProOpDB) and the University of Georgia (DOOR^2^) were used [37,38]. Listed in Table 2 are the genes with FadR binding sites identified within their promoters, the position of these genes within described transcriptional units and/or operons, and their protein names/postulated functions, as indicated by the KEGG and UniProtKB databases [39,40]. Note that several genes that had overlapping FadR/core promoter sequences (*e*.*g*., *TTHA0846*, *TTHB017*, and *TTHA0390*), were found to be downstream members of postulated operons. For these, it is unclear whether FadR is an important transcriptional regulator for controlling their expression and under what circumstances FadR has an effect on the expression of these downstream genes. However, the proposed roles for most of these genes were various enzymatic processes involved in the biosynthesis and degradation of fatty acids (3-hydroxyacyl-CoA dehydrogenase, acetyl-CoA acetyltransferase, acyl CoA dehydrogenase, fatty acid-CoA ligases, *etc*.). Most interesting was that FadR potentially regulates its own operon (*TTHA0103–TTHA0101*), something that would be expected for feedback regulation of a transcriptional repressor.

**Table 2:** Potential FadR-regulated genes.

Promoter	Operon	Gene	Role	Ratio (*fadr*Δ:wt)	Fold change	Adj. *P*-value
Y	1	*TTHA0890*	3-hydroxyacyl-CoA dehydrogenase	9775.4/375.3	26.0	3.06e-5
	2	*TTHA0891*	acetyl-CoA acetyltransferase	14762.8/426.9	34.6	1.63e-4
	3	*TTHA0892*	acyl-CoA dehydrogenase	11722/449.7	26.1	4.68e-4
Y	1	*TTHA0401*	hypothetical protein	641.7/209.9	3.1	7.34e-3
	2	*TTHA0400*	zinc-binding dehydrogenase	255/96.6	2.6	5.46e-2
Y	N	*TTHA0402*	hypothetical protein	275.2/113	2.4	5.46e-2
Y	N	*TTHA0604*	medium-chain-fatty-acid—CoA ligase	868.3/307.1	2.8	7.34e-3
Y	(2)	*TTHA0846*	metallo-beta-lactamase	923.3/161.2	5.7	4.95e-3
	3	*TTHA0845*	AsnC family transcriptional regulator	704/475.6	1.5	0.251
	4	*TTHA0844*	CAAX amino terminal protease	331.1/146.5	2.3	0.146
Y	N	*TTHA1117*	iron-sulfur protein	2793.2/678.8	4.1	1.34e-2
Y	N	*TTHA1118*	hypothetical protein	472.4/269.6	1.8	0.116
Y	1 (*7*)	*TTHB017*	medium-chain acyl-CoA ligase-related protein	2938.7/1085.4	2.7	5.46e-2
	2 *8*	*TTHB016*	gluconate 5-dehydrogenase	308.4/174.6	1.8	0.204
	3 *9*	*TTHB015*	acyl-CoA dehydrogenase	224.3/143	1.6	0.212
	4 *10*	*TTHB014*	phosphotransferase	137.6/64.9	2.1	0.365
	5 *11*	*TTHB013*	hypothetical protein	149.8/106.5	1.4	0.445
	6	*TTHB012*	phosphoglycerate mutase family protein	90.3/62.7	1.4	0.257
Y	1 (*9*)	*TTHA0390*	hypothetical protein	154.9/87.1	1.8	0.203
	2 *10*	*TTHA0391*	hypothetical protein	115.3/68.8	1.7	0.246
	3 *11*	*TTHA0392*	methylmalonyl-CoA epimerase	712.4/550.6	1.3	0.349
	4 *12*	*TTHA0393*	hypothetical protein	120.6/146.6	0.8	0.553
Y	1	*TTHA1462*	phosphoribosyltransferase	1668.7/795.4	2.1	0.296
	2	*TTHA1461*	hypothetical protein	917.5/953.7	1.0	0.965
Y	N	*TTHA1463*	long-chain fatty acid—CoA ligase	3145.6/867.1	3.6	4.04e-2
Y	1	*TTHA1143*	sensor histidine kinase	166.4/183.9	0.9	0.682
	2	*TTHA1142*	response regulator receiver domain-containing protein	121.8/62.9	1.9	0.228
	*3*	*TTHA1141*	cation-transporting ATPase	2421.4/854.7	2.8	5.46e-2
Y	1	*TTHA1144*	acetyl-coenzyme A dehydrogenase medium subunit	6247.9/1088.1	5.7	1.26e-3
	2	*TTHA1145*	electron transfer flavoprotein subunit beta	6786.9/1190.5	5.7	2.26e-3
	3	*TTHA1146*	electron transfer flavoprotein subunit alpha	4030.9/866.5	4.7	1.79e-3
Y	1	*TTHA0103*	oxidoreductase	488.6/134.8	3.6	4.67e-2
	2	*TTHA0102*	hypothetical protein	326.1/104.4	3.1	2.58e-2
	3	*TTHA0101*	TetR family transcriptional regulator, *fadR*	16.5/201.2	(0.1)	(1.26e-3)

(Operon) Number indicates gene position within an operon. Parentheses indicates FadR site is not before the first gene of an identified operon. Values in italics indicate differences between databases in their identification of operon members. (N) Single transcriptional unit, not part of an operon. (Gene) Underlined genes indicate those identified by previous investigators [7]. (Ratio) Ratio of averaged Affymetrix GeneChip signals from Δ*fadR* and wild-type strains of *T*. *thermophilus* HB8 deposited in NCBI GEO by Agari *et al*. [7]. (Fold change) Fold change in expression, Δ*fadR*:wt *T*. *thermophilus* HB8 strains.

### Validation of potential *T*. *thermophilus* FadR-regulated genes

**Fig 6:**
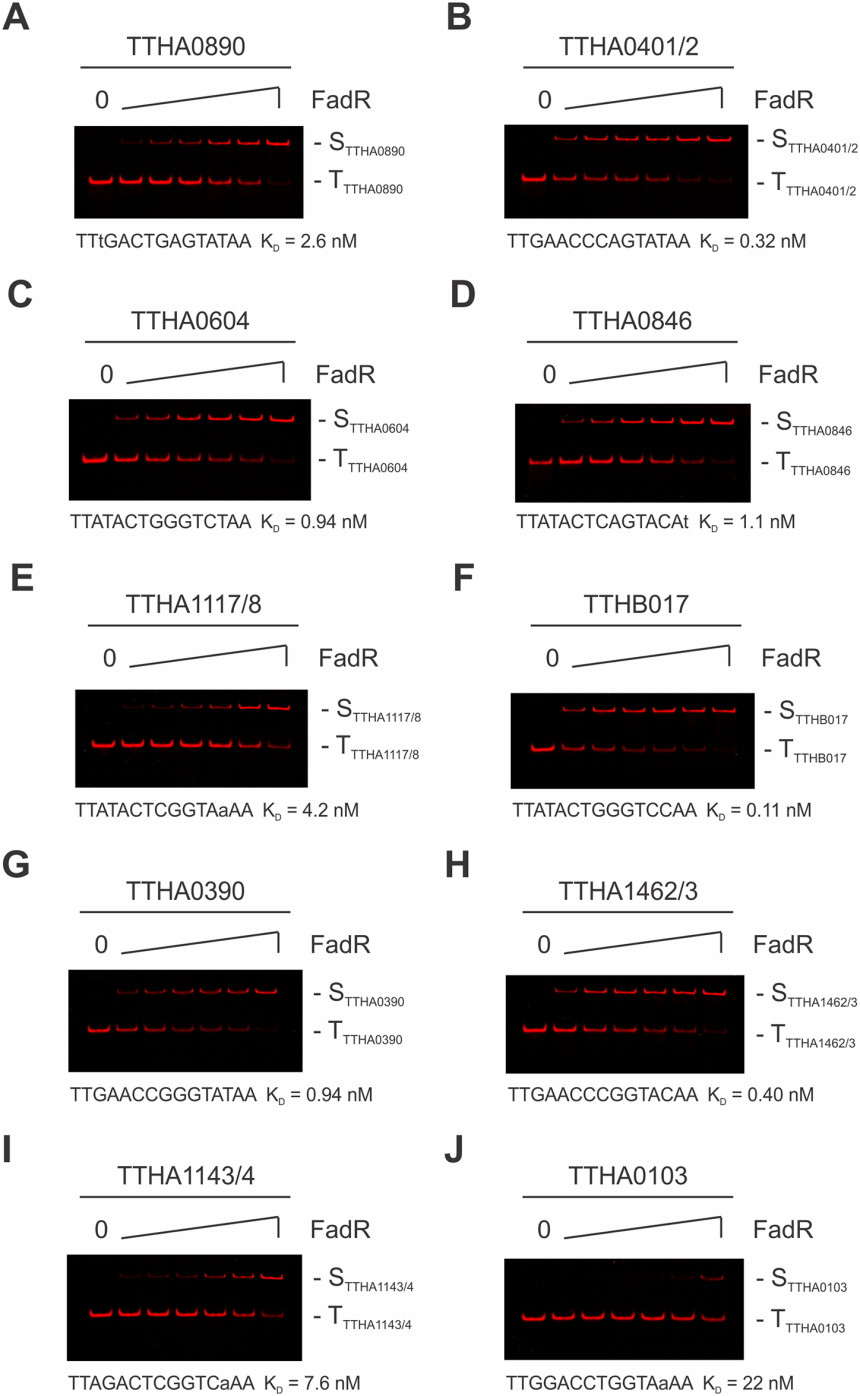
EMSA analysis of FadR-binding to REPSA-identified promoter sequences through a titration of FadR concentrations. Shown are LICOR images of IRD700-labeled DNA probes containing FadR-binding sites from the REPSA-identified promoter regions, as indicated, incubated with 0, 0.38, 0.75, 1.5, 3, 6, or 12 nM FadR protein. (S) FadR-DNA complex, (T) uncomplexed DNA. (**A**) ST2_FadR_TTHA0890 DNA. (**B**) ST2_FadR_TTHA0402 DNA. (**C**) ST2_FadR_TTHA0604 DNA. (**D**) ST2_FadR_TTHA0846 DNA. (**E**) ST2_FadR_TTHA1118 DNA. (**F**) ST2_FadR_TTHB017 DNA. (**G**) ST2_FadR_TTHA0390 DNA. (**H**) ST2_FadR_TTHA1463 DNA. (**I**) ST2_FadR_TTHA1144 DNA. (**J**) ST2_FadR_TTHA0103 DNA. Binding site sequence and K_D_ values are indicated below each panel. Lowercase nucleotides indicate mutation from consensus FadR sequence.

Having identified potential FadR-regulated genes through a process involving REPSA-identified preferred FadR-DNA binding sites and bioinformatic analyses, it is important to verify whether these gene promoters actually bind FadR and/or are regulated by FadR *in vivo*. To accomplish this, we first investigated FadR-binding to the 14 identified promoter sites by quantitative EMSA. Sequences of their DNA probes are shown in S1 Table. EMSA experiments were performed through a common range of FadR concentrations (0.38–12 nM), given that all of these probes exhibited appreciable FadR binding within this range (Fig 6). Quantitation was accomplished through a densitometric analysis of the data and an approximate FadR-DNA K_D_ determined using an equilibrium binding model and shown in Fig 6. Apparent K_D_ values ranged from 0.053 to 11 nM for these FadR binding sites, comparable to those values observed previously with the consensus sequence and mutants (Fig 4). However, they do not correlate as well with the order of sequences identified in the original FIMO analysis with some of the lower FIMO-ranked sequences exhibiting very low K_D_ (*TTHB017* and *TTHA1463*) and *vice versa* (*TTHA0890*). Such may be indicative of a limitation with the FIMO ranking algorithm.

To determine whether FadR may be involved in transcriptional regulation of these promoters, publically available gene expression data comparing mRNA levels in wild-type and FadR (*TTHA0101*)-deficient *T*. *thermophilus* HB8 were analyzed. GeneChip microarray data GSE24184 from the National Center for Biotechnology Information Gene Expression Omnibus functional genomics data repository, which compares four *T*. *thermophilus* HB8 wild-type strains and three *TTHA0101*-deficient mutant strains, was used [7,41]. The ratio in expression between averaged wild-type and *TTHA0101*-deficient samples, fold change, and adjusted *P*-values are indicated for each gene in Table 2. We found fold differences ranging from nearly 35-fold overexpression (*TTHA0891*) to near comparable levels of expression (*TTHA0393*, *TTHA1143*, *TTHA1461*) for these putative FadR-regulated genes, with most (20/34) exhibiting a two-fold or greater increase in expression when FadR was absent. More significant, adjusted *P*-value measures ranged from 3.1 x 10^−5^ to 0.97, with most of these genes (20/34) being in the top 250 of all genes identified by the GEO2R comparison (*P*-value ≤ 0.146). Taken together, these analyses strongly suggest an involvement of FadR in the regulation of the REPSA-identified gene promoters.

Additional approaches to support a role for FadR in the regulation of the REPSA-identified gene promoters were pursued through comparative genomic means. Approaches such as phylogenetic footprinting, which looks at the conservation of the orthologous FadR binding site among related species and regulon inference, identifying co-regulated orthologous operons among related species, were used [42,43]. 415-bp sequences centered on the FadR binding site and containing the promoter region (Fig 5) were used in a BLASTn search of the genomes of related organisms in the *Deinococcus-Thermus* group [44]. If orthologous regions were found, a search was made for the orthologous FadR site and its sequence conservation to the REPSA-determined consensus ascertained. We found that all of the *T*. *thermophilus* HB8 FadR binding sites were conserved in orthologous gene promoters in the highly related strain *T*. *thermophilus* HB27 (Table 3) with the exception of the *TTHB017* promoter, which is present on a plasmid not present in the HB27 strain. For more distant *Thermus* species (*T*. *aquaticus* Y51MC23, *T*. *oshimai* JL2), most all of the orthologous gene promoters had identifiable FadR binding sites, although many had lower similarity to the *T*. *thermophilus* HB8 consensus. These phylogenetic footprinting data suggest that FadR binding sequences exist in the promoter regions of orthologous genes in *Thermus* species, consistent with a role for this protein in their regulation. However, a similar analysis with more distant *Deinococcus* species (*D*. *radiodurans* R1, *D*. *deserti* VCD115, *D*. *geothermalis* DSM 11300) found no identifiable FadR sites in orthologous genes (data not shown). Such may be a reflection of the phylogenetic distance for the *Deinococcus* species compared to the *Thermus* species and/or the presence of a related FadR transcription regulatory protein with a different binding specificity. Alternatively, it may be a limitation of the algorithm used in BLASTn searches.

**Table 3:** BLASTn analysis of orthologous FadR sites.

ttj	tth	taq	tos
*TTHA0890*	*TT_C0534* | 15/15	(*TO73_1062*) | 14/15	(*Theos_1531*) | 14/15
*TTHA0401*	*TT_C0033* | 15/15	*TO73_2041* | 13/15	*Theos_1574* | 13/15
*TTHA0402*	*TT_C0034* | 15/15	*TO73_2040* | 13/15	*Theos_1573* | 13/15
*TTHA0604*	*TT_C0236* | 15/15	–	–
*TTHA0846*	*TT_C0494* | 15/15	–	*Theos_0875* | 13/15
*TTHA1117*	*TT_C0752* | 15/15	*TO73_0756* | 15/15	*Theos_1481* | 15/15
*TTHA1118*	*TT_C0753* | 15/15	*TO73_0755* | 15/15	*Theos_1482* | 15/15
*TTHB017*	–	*TO73_0923* | 10/15	*Theos_1364* | 14/15
*TTHA0390*	*TT_C0023* | 15/15	*TO73_2051* | 13/15	*Theos_1584* | 7/15
*TTHA1462*	*TT_C1098* | 15/15	*TO73_1544* | 13/15	*Theos_1668* | 13/15
*TTHA1463*	*TT_C1099* | 15/15	*TO73_1545* | 13/15	*Theos_1668* | 13/15
*TTHA1143*	*TT_C0778* | 15/15	*TO73_1042* | 14/15	*Theos_1508* | 14/15
*TTHA1144*	*TT_C0779* | 15/15	*TO73_1043* | 14/15	*Theos_1509* | 14/15
*TTHA0103*	*TT_C1901* | 15/15	(*TO73_1882*) | 13/15	(*Theos_1721*) |13/15

Organisms investigated include *Thermus thermophilus* HB8 (ttj), *Thermus thermophilus* HB27 (tth), *Thermus aquaticus* Y51MC23 (taq), and *Thermus oshimai* JL2 (tos). (15/15) The number of identical bases present in the orthologous gene FadR site. (*TO73_1062*) Genes in parentheses have FadR sites downstream of their translation start sites.

^(–)^ Orthologous sequences not found.

To better identify FadR-regulated operons in other organisms, a comparative genomics regulon inference analysis was performed using RegPredict, available *Deinococcus-Thermus* genomes, and a profile built from a training set composed of the FadR binding sequences present in the REPSA-identified promoters [45]. Additional parameters included searching for FadR profile sequences in a region –200 to +50 relative to the gene translation start site, allowing coding region overlap, operons based on a 200 bp maximum intergenic distance, and a score threshold of 4.80. This RegPredict run yielded 39 clusters of co-regulated orthologous operons (CRONs), of which 14 contained at least one *T*. *thermophilus* HB27 operon with an identified FadR binding sequence in its promoter region. This choice of focusing on *T*. *thermophilus* HB27 operon-containing CRONs was made given the high degree of similarity observed between the promoters of *T*. *thermophilus* HB8 and HB27 as found in our BLASTn analysis (Table 3). Table 4 shows the *T*. *thermophilus* HB27 operons identified as potentially being regulated by FadR, as well as operons in related organisms that may be regulated by FadR. We found that for the highest scoring CRONs (9, 2, 4, 1), many of the orthologous genes in related organisms were identified as possessing FadR-binding sites within their promoter regions. For the lower scoring CRONs, FadR-binding sites may be found in one or more organisms but most orthologous genes lack FadR-binding sites in their control regions. Similarly, while most CRONs identified orthologous, FadR-binding genes in the closely related organism *T*. *aquaticus*, many of the CRONs contained genes for which no orthologs were identified in any of the *Deinococcus* species. All in all, the RegPredict analysis was capable of identifying evidence for potential FadR regulation in orthologous genes from more phylogenetically distant organisms than those identified through a BLASTn study. Taken together, these studies support the contention that FadR has the potential to regulate homologous genes in many of these organisms.

**Table 4:** FadR clusters of co-regulated orthologous operons.

CRON	Max. score	tth	taq	dra	ddr	dge
9	5.33	*TT_C0779–0781*	3w	3s	3s	2s, 1w
2	5.28	*TT_C0752*	1s	1n	1s	1s
4	5.26	*TT_C0534–0536*	3s	1s, 1n	2w	1w, 1n
1	5.23	*TT_C0023–0025*	3s	3n	2s	3n
28	5.28	*TT_C0753*	1s	–	–	–
12	5.20	*TT_C1099*	1s	–	–	–
13	5.20	*TT_C1098–1097*	2s	–	–	–
10	5.18	*TT_C0033–0026*	2s, 6n	3n	3n	3n
8	5.11	*TT_C1901*	3s	1n	1w, 1n	1w
20	5.29	*TT_C0236–0234*	3n	2n	1n	2n
25	5.18	*TT_C0034*	–	–	–	–
3	5.06	*TT_C0778–0776*	2w, 1n	–	–	–
30	5.00	*TT_C1065–1067*	3n	–	–	–
29	4.81	*TT_C0494*	5n	1w	3n	3n

(CRON) Co-regulated orthologous operon number from the RegPredict run. (Max. Score) Measure of the similarity of candidate FadR-binding sites in the orthologous promoters compared to the position-weight matrix made by the training set. Organisms investigated include *T*. *thermophilus* HB27 (tth), *T*. *aquaticus* Y51MC23 (taq); *D*. *radiodurans* R1 (dra), *D*. *deserti* VCD115 (ddr), and *D*. *geothermalis* DSM 11300 (dge). Genes within the FadR-regulated *T*. *thermophilus* HB 27 operon are shown. For the other organisms, indicated are the numbers of orthologous genes that have strong (s), weak (w), or no (n) identified FadR sites in their promoters.

^(–)^ No orthologous operons identified.

## Discussion

Determining regulons, transcriptional regulatory networks, in organisms classically follows the approach: (1) find changes in the transcriptome between control/stress or wild type/mutant organisms, (2) identify co-regulated genes, and (3) determine common promoter sequences consistent with a regulatory transcription factor binding site. These binding sites can then be validated by physical and function means, including methods such as surface plasmon resonance and *in vitro* transcription. We have used an alternative approach: (1) determining a consensus transcription factor binding sequence by a combinatorial selection method and motif discovery software, (2) bioinformatically identify potential binding sites in the genome, and (3) describe putative regulated genes to gain insights into the regulatory network controlled by the *T*. *thermophilus* HB8 transcription factor FadR. Here validation can also be accomplished through *in vitro* and *in vivo* methods, including EMSA and qPCR. As FadR had been the subject of a prior study, our present work allows a direct comparison between the two approaches for their ability to define a regulon for this protein [7].

Using REPSA, massive parallel semiconductor sequencing, and MEME motif discovery software, we obtained the consensus 15-bp sequence 5´-TTRNACYNRGTNYAA-3´ with high significance, being present on 85% of the sequenced DNA. This palindromic sequence maps in part to the consensus sequence determined by Agari *et al*., 5´-TTANACT–(N_6–7_)–ARNNNAR-3´, particularly its 5´-most end [7]. Using EMSA, we determined the dissociation constant for FadR binding to the consensus sequence 5´-TTGGACTTAGTCCAA-3´ to be K_D_ = 0.17 nM. This value is 500-fold lower than that found by Agari *et al*. for the FadR binding sequence in the *TTHA0890* promoter (K_D_ = 90 nM) but could reflect differences in the two experimental methods used, given that we obtained a K_D_ = 2.6 nM using EMSA for the *TTHA0890* site. As FadR is structurally a TetR-family transcriptional repressor protein, it would be expected to bind a palindromic DNA sequence as a homodimer, consistent with the 15-bp consensus sequence we identified. The role of the 3´ heptameric sequence in the Agari *et al*. consensus sequence remains as yet uncertain but its appearance may reflect differences in the motif discovery software employed. This is supported by the observation that a MEME analysis of the nine FadR-regulated promoter regions identified by Agari *et al*. yields a 15-bp pseudopalindromic sequence 5´-TTkkACYsRGTMYAA-3´ with an *E*-value of 4.8e-021. This sequence is actually quite similar to the 15-bp palindromic consensus sequence we have identified but lacks the 3´ heptameric sequence in question. Nonetheless, a position weight matrix defined by thousands of sequences can be considerably more significant than one defined by 10 sequences or less. Ultimately, this can have an impact on subsequent analyses, *e*.*g*., identification of binding sites within an organism’s genome.

We used the motif scanning software FIMO to identify putative FadR binding sites within the *T*. *thermophilus* HB8 genome. Using quality cutoffs to filter for the most promising sites, we found 16 genomic FadR-binding sequences, 14 of which were located within potential promoter regions. These promoter regions were further characterized for the locations of potential core promoter elements (–35 box, –10 box, +1 site). In each case, FadR mapped to a site within or overlapping these core elements, as would be expected for a transcriptional repressor protein. Notably, nine of the 14 promoters we identified were previously identified by Agari *et al*. (see Fig 5, underlined genes), suggesting that both approaches converge on a similar set of genes [7]. In addition, while differences exist between the two approaches regarding the binding sites recognized by FadR (Fig 5, compare yellow highlighting and italicized bases), there was a great deal of similarity between the core promoter elements identified by each (Fig 5, compare blue highlighting and underlined bases). FadR binding to these promoter sites was independently analyzed by EMSA and found to have dissociation constants in the range of 0.11 to 22 nM, all reasonable affinities for a prokaryotic transcription factor to its recognition site. Taken together, these data strongly support the hypothesis that FadR could regulate these genes.

Our REPSA-initiated approach identified five promoters not previously described, those upstream of *TTHA0402*, *TTHA1118*, *TTHA0390*, *TTHA1462*, and *TTHA1143*. Curiously, four of these are members of bidirectional promoters (*TTHA0401/2*, *TTHA1117/8*, *TTHA1462/3*, and *TTHA1143/4*), one of whose members had been previously identified as being FadR-regulated [7]. Given the compact nature of the *T*. *thermophilus* HB8 genome, one might expect some degree of co-regulation among bidirectional promoters [46]. This we attempted to validate using available gene expression data from experiments with wild-type and *fadR*Δ mutants [7,41]. Those nine genes identified in both approaches demonstrated 26 to 2.7-fold (median 3.6-fold) increase in expression in the absence of FadR, while those additional genes we identified by the REPSA-based approach had expression increases in the 2.4 to 0.9-fold (median 1.8-fold) range, appreciably less. This is understandable, given that the genes with the greatest changes in expression were those identified in the transcriptome-based approach. Reasons for the observed reduced levels of induction among the lower third of genes may be due in part to intrinsically low levels of expression for these genes (*e*.*g*., *TTHA0402*, *TTHA0390*, and *TTHA1143*) or the possibility of multiple levels of repression. Thus, while the reduced levels of induction do not completely exclude the possibility that these genes may be regulated by FadR under certain conditions, they do tend to diminish the confidence in their being *bona fide* FadR-regulated promoters.

It has been suggested that comparative genomics analyses can provide additional evidence supporting a role for a transcription factor and cognate binding site in regulating the expression of particular genes. Thus, phylogenetic footprinting and a regulon inference analysis were performed with our FadR-identified sequences. Phylogenetic footprinting using BLASTn found conservation of FadR binding sites among orthologous gene promoters in closely related organisms, further supporting that these sites play a role in their gene regulation. On the other hand, the regulon inference analysis performed with RegPredict has the potential to discover additional promoters. Thus, while RegPredict identified 13 of the 14 *T*. *thermophilus* HB27 promoters we had previously identified in orthologous *T*. *thermophilus* HB8 gene promoters (compare Tables 4 and 3), it also found an additional potential FadR-regulated promoter upstream of the operon *TT_C1065–TT_C1067*. The genes in this operon are orthologous to the *T*. *thermophilus* HB8 genes *TTHA1430*, *TTHA1431*, and *TTHA1432*, which are thought to encode a long chain fatty acid-CoA ligase and two hypothetical proteins, respectively. A review of the available gene expression data finds these genes to be induced 5.4, 1.6, and 1.1-fold in *fadR*Δ mutant strains [7,41]. Taken together, these data suggest that at least *TTHA1430* may be a FadR-regulated gene. Interestingly, the FadR site upstream of *TTHA1430* was identified in our FIMO search, albeit with a significance (*P*-value = 1.56e-06, *Q*-value = 0.235) slightly lower than the cutoff we employed. This suggests that we may have to decrease the threshold for acceptable sites in future FIMO searches to better capture potential regulated genes. It also demonstrates the utility of incorporating a regulon inference analysis into our REPSA-initiated approach for regulon discovery.

## Materials and methods

### Oligonucleotides

Oligonucleotides used in this study were synthesized by Integrated DNA Technologies and are listed in S1 Table. Double-stranded DNA was prepared from single-stranded oligonucleotides by PCR using New England Biolabs (NEB) *Taq* DNA polymerase and standard reaction conditions as indicated by the manufacturer. REPSA selection template ST2R24 was initially prepared with minimal PCR cycles (6) to ensure that the resulting product was primarily duplex DNA with fully annealed randomized cassette regions.

### Expression and purification of FadR protein

Plasmid pET11a-ttFadR, which contains the *T*. *thermophilus TTHA0101* (*fadR*) gene under the control of a T7 promoter in the *E*. *coli* expression vector pET-11a, was obtained from the RIKEN Structural Biology Laboratory and was the generous gift of Dr. Akeo Shinkai [7]. Bacterial transformation, FadR expression and purification followed the procedures used previously for *T*. *thermophilus* HB8 SbtR [30]. Protein concentration was determined using a Bio-Rad protein assay and estimated at 0.7 mg/ml. Protein purity was investigated by SDS-PAGE using Bio-Rad TGX Stain-free gels and stain-free imaging technology or Agilent P200 ScreenTape assays (S1 Fig). The stock FadR solution used in this study was estimated as containing no greater than 30 μM FadR monomer or 15 μM FadR_2_, the dimeric form presumed to bind DNA.

### REPSA selection

REPSA selections with FadR followed the procedures used previously with SbtR, with the exception that the IISRE FokI was used in Rounds 1–3 and BpmI in Rounds 4 and 5.

### Electrophoretic mobility shift assays

Electrophoretic mobility shift assays (EMSA) followed the procedures used previously with SbtR [30]. Quantitative EMSA with defined DNAs followed a two-step protocol. (1) An initial EMSA was performed with a 10-fold serial dilution of FadR, to roughly determine that concentration of FadR that would yield a 50:50 distribution of free DNA and FadR-DNA complex. (2) A second EMSA was performed with two-fold serial dilutions of FadR bracketing the aforementioned 50:50 concentration, to better aid in its determination. Band intensities from the second EMSA were determined using LICOR Image Studio software. Dissociation constants were determined using a standard binding equilibrium equation (K_D_ = [DNA_free_]*[FadR_free_]/[FadR-DNA_complex_] where [DNA_free_] = [DNA_total_] * fraction unbound, [FadR-DNA_complex_] = [DNA_total_] * fraction in complex species, and [FadR_free_] = [FadR_total_]–[FadR-DNA_complex_]) with values for free DNA and DNA-FadR complex concentrations being obtained from the image analysis software.

### Massive parallel sequencing of REPSA-selected DNAs

Amplicon library preparation, Ion PGM individual sequencing particle (ISP) preparation, Ion PGM semiconductor sequencing, and Ion torrent sever sequence processing were all performed as previously described [30]. A fastq file of the Ion PGM sequencing data (S1 Text) and a processed sequencing data file suitable for MEME analysis (S2 Text) are provided in Supporting Information.

### Bioinformatics

Bioinformatics, including sequence data processing, duplicate finding, motif discovery, motif scanning, promoter identification, and operon identification were performed using the software, websites, and workflows previously described [30]. For our FIMO analysis, a stringent threshold for *P*-values (7.0e-07) and *Q*-values (0.14) was used in this study, based on prior work with related REPSA-identified transcription factor binding sites. Additional bioinformatic gene expression analyses were performed using data from the National Center for Biotechnology Information Gene Expression Omnibus website (https://www.ncbi.nlm.nih.gov/geo/) [41]. Expression levels generated from processed data were obtained from their corresponding series matrix text file. A ratio of averaged levels for mutant strains compared to the wild-type control strains was made, thereby permitting the calculation of a fold change value for each gene. Data sets were also compared using NCBI GEO2R (https://www.ncbi.nlm.nih.gov/geo/geo2r/), thereby allowing an assessment of the statistical significance of the comparison. Significance levels (*P*-values) were adjusted to correct for potential false positives using the Benjamini & Hochberg false discovery rate method [47]. Adjusted *P*-values are shown.

For our comparative genomics studies, nucleotide BLAST sequence alignment searches for FadR binding sites in orthologous gene promoters were performed using the NCBI BLASTn website (https://blast.ncbi.nlm.nih.gov/Blast.cgi), discontiguous megablast BLASTn algorithm, and default parameters [44]. For each identified promoter, the 415-bp sequence centered on the *T*. *thermophilus* HB8 FadR binding site (see Fig 5) served as query and the nucleotide collection (nr/nt) sequence databases for organisms *Thermus thermophilus* HB8 (taxid:300852), *Thermus thermophilus* HB27 (taxid:262724), *Thermus aquaticus* Y51MC23 (taxid:498848), *Deinococcus radiodurans* R1 (taxid:243240), *Deinococcus deserti* VCD115 (taxid:546414), and *Deinococcus geothermalis* DSM 11300 (taxid:319795) served as search sets. The presence of FadR binding sites in individual alignments was performed using the intrinsic browser find function while mapping these sites to specific gene promoters was performed using the BLASTn-embedded Nucleotide Graphics function.

Comparative genomics regulon inference analysis was performed using the RegPredict webserver (http://regpredict.lbl.gov/regpredict/), selecting the full set of *Deinococcus-Thermus* genomes, and determining regulon inference by known PWM using a training set composed of the putative FadR binding sites identified in *T*. *thermophilus* HB8 promoters (Fig 5) [45]. Default profile parameters were used with the exception of the target positions ranging from –200 to +50, to include possible misidentified translation start sites. Output was then presented in a tabular format with strong FadR-regulated operons in the closely related *T*. *thermophilus* HB27 genome explicitly identified and clusters of co-regulated orthologous operon member genes from other organisms indicated with a simplified measure: number of orthologous genes under strong (s), weak (w), or no (n) postulated FadR regulation. A negative sign (–) indicated no orthologous operons were identified.

## Supporting information

S1 Fig Expression and purification of FadR protein. (PDF)

S1 Table Oligonucleotides. (PDF)

S1 Text REPSA Round 5 sequences, fastq format. (FASTQ)

S2 Text REPSA Round 5 sequences, Sequencing1.java processed. (TXT)
