# Supplementary material for: Identification and characterization of preferred DNA-binding sites for the Thermus thermophilus transcriptional regulator FadR
Source: PLoS One. 2017 Sep 13;12(9):e0184796. doi: 10.1371/journal.pone.0184796 (PMC5597230; doi:10.1371/journal.pone.0184796)
Supplement: S1 Fig — (PDF) [file pone.0184796.s001.pdf]

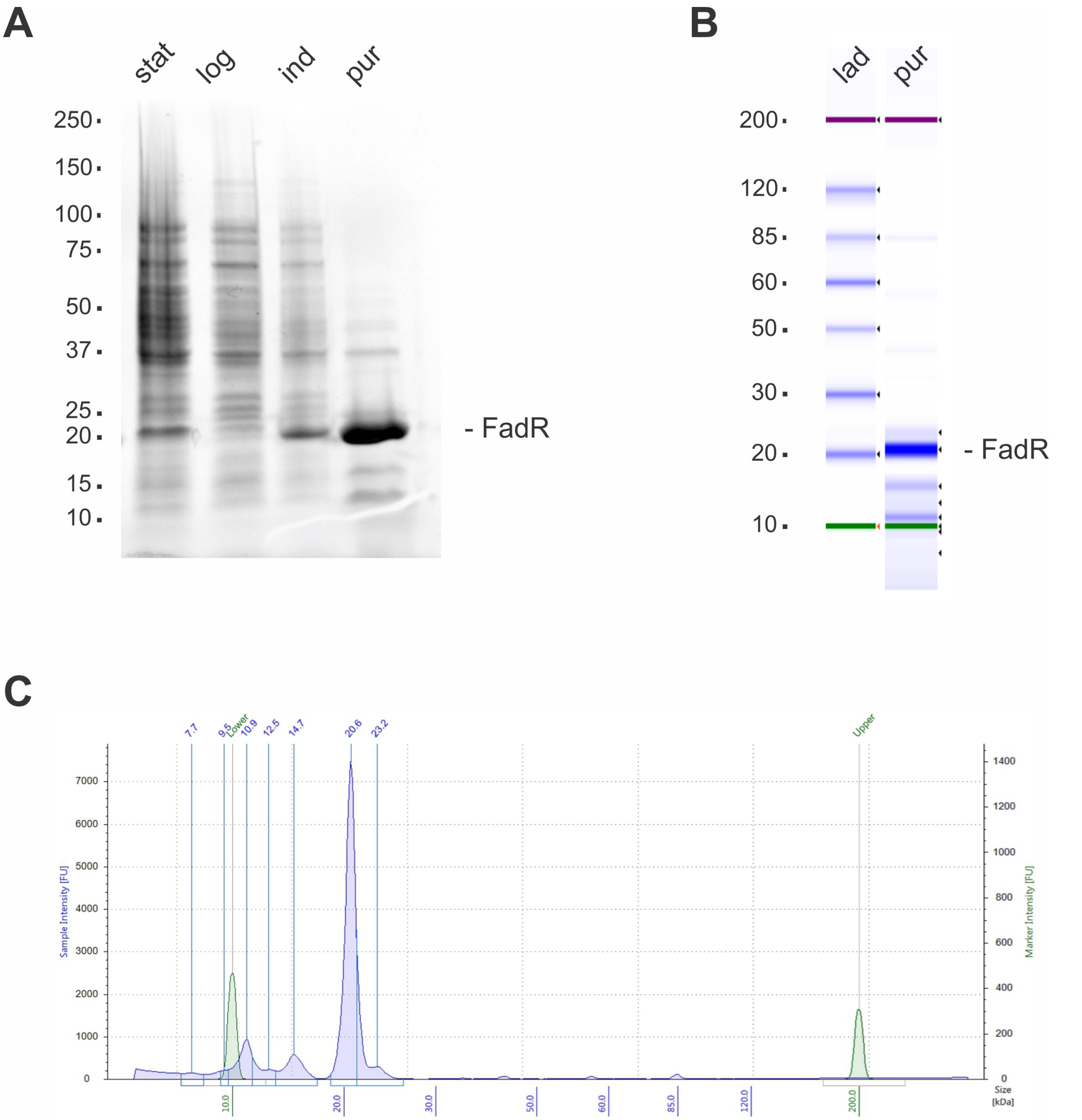

S1 Fig. Expression and purification of FadR protein. (**A**) Shown is a Bio-Rad 4-20% SDS-PAGE TGX Stain-Free gradient gel onto which was loaded whole cell extracts or partially purified fractions containing FadR protein. Lanes shown left to right: (stat) 50 µg whole cell extract from stationary growth *E coli* BL21(DE3) bacteria containing the plasmid pET11a-fadR, (log) 20 µg whole cell extract from the stationary growth bacteria following logarithmic growth

for 2.5 h, (ind) 10 μg whole cell extract from the logarithmic growth bacteria following IPTG-induction for 5 h, (pur) 35 μg purified FadR protein. The location of molecular weight standards is indicated at the left of the figure. (**B**) Gel representation of TapeStation P200 data. Lanes shown left to right: (lad) P200 ladder, (pur) 0.35 μg purified FadR protein. (**C**) Electropherogram of TapeStation P200 FadR data.
