# Supplementary material for: Identification and characterization of preferred DNA-binding sites for the Thermus thermophilus transcriptional regulator FadR
Source: PLoS One. 2017 Sep 13;12(9):e0184796. doi: 10.1371/journal.pone.0184796 (PMC5597230; doi:10.1371/journal.pone.0184796)
Supplement: S1 Table — (PDF) [file pone.0184796.s002.pdf]

S1 Table. Oligonucleotides.

Name	Sequence	Length	Purif.	Use
ST2R24	CTAGGAATTCGTGCAGAGGTGAATNNNNNNNNNNNNNNNNNNNNNNNNNTTACCATCCCTCCAGAAGCTTGGAC	73	PAGE	REPSA selection template precursor
ST2L	CTAGGAATTCGTGCAGAGGTGAAT	24	Desalt	PCR primer
ST2R	GTCCAAGCTTCTGGAGGGATGGTAA	25	Desalt	PCR primer
IRD7_ST2R	/5IRD700/GTCCAAGCTTCTGGAGGGATGGTAA	25	HPLC	5′ IRDye 700-modified PCR primer
A_BC01_ST2R	CCATCTCATCCCTGCGTGTCTCCGACTCAGCTAAGGTAACGATGTCCAAGCTTCTGGAGGGATG	64	PAGE	Fusion PCR primer
trP1_ST2L	CCTCTCTATGGGCAGTCGGTGATCTAGGAATTCGTGCAGAGGTGA	45	PAGE	Fusion PCR primer
A_uni	CCATCTCATCCCTGCGTG	18	Desalt	PCR primer
trP1_uni	CCTCTCTATGGGCAGTCGG	19	Desalt	PCR primer
Bio_ST2R	/5BiodT/GTCCAAGCTTCTGGAGGGATG	22	HPLC	5′ biotin-modified PCR primer
ST2_FadR_R5_wt	AGGAATTCGTGCAGAGGTGAATTTGGACTTAGTCCAATTACCATCCCTCCAGAAGCTTGG	60	Desalt	FadR consensus DNA probe precursor
ST2_FadR_R5_m1	AGGAATTCGTGCAGAGGTGAATGTGGACTTAGTCCAATTACCATCCCTCCAGAAGCTTGG	60	Desalt	FadR mutant 1 DNA probe precursor
ST2_FadR_R5_m2	AGGAATTCGTGCAGAGGTGAATTGGGACTTAGTCCAATTACCATCCCTCCAGAAGCTTGG	60	Desalt	FadR mutant 2 DNA probe precursor
ST2_FadR_R5_m3	AGGAATTCGTGCAGAGGTGAATTTCGACTTAGTCCAATTACCATCCCTCCAGAAGCTTGG	60	Desalt	FadR mutant 3 DNA probe precursor
ST2_FadR_R5_m4	AGGAATTCGTGCAGAGGTGAATTTGCACTTAGTCCAATTACCATCCCTCCAGAAGCTTGG	60	Desalt	FadR mutant 4 DNA probe precursor
ST2_FadR_R5_m5	AGGAATTCGTGCAGAGGTGAATTTGGGCTTAGTCCAATTACCATCCCTCCAGAAGCTTGG	60	Desalt	FadR mutant 5 DNA probe precursor
ST2_FadR_R5_m6	AGGAATTCGTGCAGAGGTGAATTTGGAATTAGTCCAATTACCATCCCTCCAGAAGCTTGG	60	Desalt	FadR mutant 6 DNA probe precursor
ST2_FadR_R5_m7	AGGAATTCGTGCAGAGGTGAATTTGGACATAGTCCAATTACCATCCCTCCAGAAGCTTGG	60	Desalt	FadR mutant 7 DNA probe precursor
ST2_FadR_TTHA0890	AGGAATTCGTGCAGAGGTGAATTTTGACTGAGTATAATTACCATCCCTCCAGAAGCTTGG	60	Desalt	TTHA0890 FadR-site DNA probe precursor
ST2_FadR_TTHA0402	AGGAATTCGTGCAGAGGTGAATTTGAACCCAGTATAATTACCATCCCTCCAGAAGCTTGG	60	Desalt	TTHA0402 FadR-site DNA probe precursor

ST2_FadR_TTHA0604	AGGAATTCGTGCAGAGGTGAATTTATACTGGGTCTAATTACCATCCCTCCAGAAGCTTGG	60	Desalt	TTHA0604 FadR-site DNA probe precursor
ST2_FadR_TTHA0846	AGGAATTCGTGCAGAGGTGAATTTATACTCAGTACATTTACCATCCCTCCAGAAGCTTGG	60	Desalt	TTHA0846 FadR-site DNA probe precursor
ST2_FadR_TTHA1118	AGGAATTCGTGCAGAGGTGAATTTATACTCGGTAAAATTACCATCCCTCCAGAAGCTTGG	60	Desalt	TTHA1118 FadR-site DNA probe precursor
ST2_FadR_TTHB017	AGGAATTCGTGCAGAGGTGAATTTATACTGGGTCCAATTACCATCCCTCCAGAAGCTTGG	60	Desalt	TTHB017 FadR-site DNA probe precursor
ST2_FadR_TTHA0390	AGGAATTCGTGCAGAGGTGAATTTGAACCGGGTATAATTACCATCCCTCCAGAAGCTTGG	60	Desalt	TTHA0390 FadR-site DNA probe precursor
ST2_FadR_TTHA1463	AGGAATTCGTGCAGAGGTGAATTTGAACCCGGTACAATTACCATCCCTCCAGAAGCTTGG	60	Desalt	TTHA1463 FadR-site DNA probe precursor
ST2_FadR_TTHA1144	AGGAATTCGTGCAGAGGTGAATTTAGACTCGGTCAAATTACCATCCCTCCAGAAGCTTGG	60	Desalt	TTHA1144 FadR-site DNA probe precursor
ST2_FadR_TTHA0103	AGGAATTCGTGCAGAGGTGAATTTGGACCTGGTAAAATTACCATCCCTCCAGAAGCTTGG	60	Desalt	TTHA0103 FadR-site DNA probe precursor

(N) Random nucleotides. Length is in nucleotides.
